# Testing Theory-Enhanced Messaging to Promote COVID-19 Vaccination: Results from a Randomized Controlled Trial

**DOI:** 10.21203/rs.3.rs-6472442/v1

**Published:** 2025-05-02

**Authors:** Rachael Piltch-Loeb, Yanhan Shen, Sasha Fleary, McKaylee Robertson, Josefina Nunez, Kate Penrose, Jenna Sanborn, Surabhi Yadav, Avantika Srivastava, Denis Nash, Angela Parcesepe

**Affiliations:** CUNY Graduate School of Public Health and Health Policy; CUNY Graduate School of Public Health and Health Policy; CUNY Graduate School of Public Health and Health Policy; City University of New York; CUNY Graduate School of Public Health and Health Policy; CUNY Graduate School of Public Health and Health Policy; CUNY Graduate School of Public Health and Health Policy; CUNY Graduate School of Public Health and Health Policy; CUNY Graduate School of Public Health and Health Policy; City University of New York Graduate School of Public Health and Health Policy; UNC Chapel Hill

**Keywords:** COVID-19 vaccine, Attitudinal Inoculation, CBT-kernels, Public health communication

## Abstract

Uptake of the COVID-19 vaccine has been low in the US (~ 22% among adults in 2023 − 24^1^) despite ongoing public health recommendations. This has been linked to many factors including pandemic fatigue, reduced risk perception, dis/misinformation, and recently, symptoms of depression and anxiety. Novel communication and messaging strategies are one potential approach to promote vaccine uptake. This randomized control trial tests two communication-based approaches compared to standard public health messaging on vaccine uptake in a cohort of adult US residents. We completed a 3-arm, parallel-group, assessor-blinded stratified-randomized trial between April-15–2024 and May-2–2024. Eligible individuals were ≥ 18 years old who: 1)had received at least one dose of the COVID-19 vaccine, but, 2)had not received COVID-19 vaccine doses since September-11–2023, and 3)had not been infected with SARS-CoV-2 in the past three months. We purposively sampled eligible individuals with and without symptoms of anxiety and depression. Participants were randomly allocated to: 1) attitudinal inoculation intervention; 2) CBT-kernels intervention; or 3) standard public health messaging intervention. At four-week follow up, these groups showed no meaningful differences in uptake (CBT- kernels:1.6% [95%CI:0.4–2.8]; Inoculation:0.9% [95%CI:0.0–1.8]; and Standard:1.3% [95%CI:0.3–2.4]) or level of vaccine willingness. Successful efforts to increase uptake of the COVID-19 vaccine via theory-enhanced messaging remain elusive.

## Introduction

Within the United States, vaccination for the updated COVID-19 vaccine remains suboptimal. Despite 81% of the population receiving at least one dose of any COVID-19 vaccine since 2020, as of June 8th, 2024, only 22% of adults and 14.9% of children under 17 years old had received the latest vaccine for the 2023–2024 season^1^. In the past year, the proportion of persons who did not intend to get a COVID-19 vaccination increased from 37% in September 2023 to 43% in June 2024, despite ongoing COVID-19 transmission, illness, and mortality^2,3^.

There is limited evidence on the effectiveness of communication-based interventions designed to address vaccine hesitancy, and such studies rarely include vaccine uptake as an outcome^4,5^. In addition, little is known about the extent to which effectiveness of vaccine uptake interventions differs among those with and without mental health symptoms. The COVID-19 pandemic has triggered a sharp rise in anxiety and depression symptoms, fueled in part by social isolation, economic instability, and disruptions to daily life^6,7^. Mental health symptoms not only increase the risk of severe COVID-19 outcomes^8,9^ but are also linked to a lower likelihood of receiving a COVID-19 vaccine^10,11^. Hence, there is a critical need to address COVID-19 vaccine uptake in this population.

Novel messaging strategies may be warranted to promote vaccine uptake and reduce vaccine hesitancy. ‘Attitudinal inoculation’ offers a promising approach, as a brief, scalable strategy that utilizes the power of narrative, values, and emotion to bolster resistance to misinformation and reduce vaccine hesitancy^12,13^. A quasi-experimental trial tested the efficacy of attitudinal inoculation videos to enhance COVID-19 vaccine acceptance- ‘Inoculated’ participants showed an improved ability to recognize rhetorical strategies used in misinformation, were less likely to subsequently share false information, and exhibited greater willingness to get vaccinated^14^. Though this approach has been shown to decrease COVID-19 *vaccine hesitancy* among US adults, the extent to which this approach increases COVID-19 *vaccination* remains unknown.

Given the lower rates of vaccine uptake among adults with mental health disorders and the likely effect of mental health symptomatology on vaccine uptake, an even more tailored approach may be warranted for adults with symptoms of depression and anxiety. In this context, Embry^15^ suggested the use of “evidence-based kernels” as a public health approach to improve mental health through behavioral vaccines, referring to the repetitive use of a unit of behavioral influence such as positive reinforcement. Other authors have suggested integrating COVID-19 public health measures with evidence-based psychological treatments such as cognitive behavioral therapy (CBT) to improve population mental health during the pandemic^16^. CBT is used to build problem-solving skills and reduce symptoms by challenging distorted cognitions, addressing overwhelming emotions, and changing maladaptive behavior^16^. Applying this to vaccine misinformation and hesitancy in individuals with symptoms of anxiety and depression may serve as a behavioral vaccine that could result in the adoption of COVID-19 prevention measures. To our knowledge, this approach has not been formally assessed.

Given the potential value of novel communication approaches to promote vaccine uptake, this study aims to fill research gaps by testing attitudinal inoculation and CBT-kernels messaging approaches on vaccine uptake among US residents. The specific aims of this study are to compare the effect of a) attitudinal inoculation versus standard messaging and b) CBT-kernels messaging versus standard messaging on 4-week COVID-19 vaccination and COVID-19 vaccine willingness; and to compare the effect of each messaging strategy on COVID-19 vaccine uptake among individuals with and without moderate or severe symptoms of anxiety or depression.

## Methods

### Trial Design

In a 3-arm, parallel-group, assessor-blinded, stratified-randomized trial, participants were enrolled based on the following eligibility criteria: aged 18 years or older, having received at least one dose of the COVID-19 vaccine but no doses since September 11, 2023, and no SARS-CoV-2 infection in the three months prior to the study start. We focused on individuals with at least one prior dose—the “moveable middle”—who show some willingness to vaccinate but remain hesitant about additional doses and may be more responsive to a brief video intervention than the unvaccinated. Our team’s prior research informed the trial design, and identified safety and efficacy as their main concerns^17^.

Eligible individuals were randomly allocated to one of three interventions: 1) attitudinal inoculation, 2) CBT-kernels or 3) standard public health messaging. Recruitment and randomization started on April 15, 2024 and continued on a rolling basis until May 02, 2024. Outcome assessment at 4-week follow-up started on May 13, 2024 and was completed by June 13, 2024. After consenting, enrolled participants were randomly assigned to one of three arms at a ratio of 1:1:1. Each arm was also stratified by presence or absence of moderate to severe anxiety or depression symptoms, defined by scoring ≥10 in the Patient Health Questionnaire-8^18^ or GAD-7^19^ at any point between December 2022 and December 2023.

### Recruitment

Individuals were recruited from the CHASING COVID cohort, a well-characterized, community-based sample of geographically and socio-demographically diverse adults aged 18 years and older who reside in the US or US territories and enrolled in the cohort between March 28, 2020 and August 21, 2020.

Details of recruitment and follow-up have been described elsewhere^20^. Briefly, participants in the cohort have completed, approximately quarterly, online assessments related to health and behaviors, SARS-Cov-2 infection history, COVID-19 symptoms, and COVID-19 vaccination status.

### Informed Consent

The study received approval from the Institutional Review Boards of the City University Of New York (CUNY) Graduate School of Public Health and Health Policy (New York, NY, USA) (protocol NCT06119854) and followed the Consolidated Standards of Reporting Trials (CONSORT) social and psychological interventions (SPI) reporting guidelines^21,22^. Informed consent forms were completed via web browser on participants’ computers or mobile devices at baseline and periodic follow-up assessments. The trial was pre-registered at: https://clinicaltrials.gov/study/NCT06119854

### Participants

Participant eligibility criteria at the time of study enrollment (April-June 2024) were as follows: (1) aged 18 and older, (2) able to read in English, (3) current residence in the US, (4) completed at least one survey as part of the CHASING COVID Cohort study between December 7, 2022 and December 22, 2023, and (5) undervaccinated, defined as having received at least one dose of the COVID-19 vaccine, but not an updated COVID-19 vaccine dose since September 11, 2023. Participants were excluded if they (1) had a SARS-CoV-2 infection in the past three months, (2) had never received *any* dose of a COVID-19 vaccine, or (3) were flagged for fraudulent behavior. Fraudulent participants were identified as showing evidence of duplicate or inconsistent contact information, suspicious response times, repeated enrolment attempts, or fraudulent flags raised from other CHASING COVID-based studies. Further details of participant characteristics can be found in Appendix A.

Intervention delivery and data collection occurred fully online. Participants completed an online survey at enrollment and at 4-weeks following enrollment.

### Intervention and Control Conditions

Participants viewed one of three one-minute videos. The video content of the experimental arms was developed using formative mixed-methods research, including a pre-trial survey with CHASING COVID participants on vaccine perceptions and qualitative interviews with participants reporting anxiety or depression in earlier questionnaires. This process revealed a common concern about vaccine effectiveness as a broader “meta-narrative” across participants^17^. Additionally, those with anxiety or depressive symptoms were more likely to cite not “making time” as a reason for being behind on vaccination- a theme later incorporated into the CBT-kernels condition.

Specifically: 1) The inoculation video addressed concerns about vaccine effectiveness, focused on bolstering resistance to mis/disinformation. 2) The CBT-kernels video focused on addressing barriers to vaccination, specifically making time to get vaccinated. It focused on cognitive reframing—highlighting how individuals might selectively focus on information that reinforces vaccine-related anxiety, challenging those beliefs, and encouraging problem-solving around making time to get vaccinated. 3) The standard public health messaging arm was a brief video adapted from existing public health PSAs, with no inoculation or CBT-kernels elements.

The videos were professionally created in collaboration with Long Story Short, a production company with experience in PSA creation that has worked with local, national, and global public health organizations. Our research team worked closely with the production team throughout the process, iterating on scripts, refining takes, selecting actors, and providing detailed feedback to ensure clarity and consistency. The research team included experts in mental health disorders, COVID-19 surveillance, infection and transmission, vaccine hesitancy and uptake, public health emergency preparedness and response, and risk communication. A member of the research team was present on set during filming to oversee production and ensure fidelity to our intended messaging. By creating all videos within the same production process, we maintained control over factors such as tone, format, duration, and overall presentation, reducing the risk of unintended differences that could influence participants’ responses. This approach allowed us to isolate the effects of the different messaging strategies while ensuring that all videos were aligned in terms of quality and delivery. A description and scripts of these videos can be found in Appendix D.

After receiving the brief digital intervention, participants received two reminders to get vaccinated by text or email on the first and third days after completing the baseline assessment. Messages were tailored to each arm and included a link to a COVID-19 vaccine locator. Their content can be found in Appendix A.

### Stratified Randomization Procedure and Masking

#### Sequence Generation

We used a two-stage procedure for randomization. First, participants eligible for recruitment were stratified into two groups based on the presence or absence of symptoms of anxiety or depression reported at any point between December 2022 and December 2023. Second, participants in both strata were randomized to one of the three intervention arms upon being enrolled. Thus, participants were randomized separately according to symptoms of anxiety or depression.

#### Masking

Participants and two team members involved in study operations and study implementation (AS and JN) were not blinded to study assignments. All other study team members, including investigators and the data analyst (JS), were blinded to study assignments.

### Measures

#### Intervention Implementation (Adherence) Measure

The survey was programmed to require participants to remain on the video page for at least the duration of the video. However, time spent on the webpage doesn’t necessarily reflect attentiveness, hence participants were also asked “Did this video hold your attention?” with options “Yes, it held my attention” and “No, it did not hold my attention / I did not watch the video.”

#### Primary Outcome: Receipt of COVID-19 Vaccine Dose at 4 Weeks

At the 4-week survey, participants were asked, “Have you received a COVID-19 vaccine dose since {date of intervention}?” Participants who responded “yes” were considered vaccinated; otherwise, they were not.

#### Secondary Outcome: Vaccine Willingness at 4 Weeks

At the 4-week survey, participants who had not received an additional dose of the vaccine were asked, “How willing are you to receive another COVID-19 vaccine dose?” with response options: “Very willing”, “Somewhat willing”, “Not willing”, or “Don’t know”. Only participants who responded ‘Very willing’ were defined as vaccine willing.

##### Pre-trial vaccine willingness.

After enrollment, but before randomization into an intervention arm, participants were asked, “How willing are you to receive another COVID-19 vaccine dose?” with response options: “Very willing”, “Somewhat willing”, “Not willing”, or “Don’t know”.

##### Post-trial vaccine willingness:

Immediately after the intervention, participants were asked: “How likely are you to make time to get a vaccine in the next month?” with response options: “Very likely,” “Somewhat likely,” “Not likely,” and “Don’t know/not sure.” They were also asked: “Are you planning to make an appointment to get the COVID-19 vaccine in the next month?” with options: “Already made an appointment”, “Planning”, “Not planning”, “Don’t know/not sure”. These questions were asked again in the 4-week follow-up survey, but only among participants who had not received an additional vaccine dose during that time. For participants with missing follow-up data (N=56), responses from the immediate post-intervention survey were used as imputed values.

#### Post-hoc Stratification Variables

Post-hoc stratification analyses were performed to assess differences in primary and secondary outcomes based on susceptibility to severe COVID-19, worry about COVID-19, and pre-trial perceptions of vaccine efficacy. Further details regarding the measurement of these stratification variables were provided in Appendix A.

### Statistical Analysis

#### Statistical Analysis

All statistical analyses were performed blind to study arm allocation. We present frequencies and summaries of characteristics by treatment assignment. Standardized mean differences (SMD) were reported. For post-randomization characters, Chi-squared or Fisher’s exact tests were used to compare proportions.

Primary analyses were performed under the intention-to-treat principle, including all participants who underwent randomization. Strict intention-to-treat analysis is hard to achieve for two reasons: missing outcomes for participants and protocol non-adherence. For the primary analysis, we used multiple imputation for those lost to follow-up at some point after randomization (as recommended by the CONSORT statement^23^), which has the benefit of including all randomized participants. As a sensitivity analysis, we assessed loss to follow-up assumptions under (1) multiple imputation and (2) protocol non-adherence (see Appendix).

For the primary outcome, we generated risk ratios using a robust Poisson regression model and generated risk differences. For the secondary outcome of vaccine willingness, we generated risk ratios using a Poisson regression model. The model for the overall effect estimation included the randomization arms and a term for the presence or absence of symptoms of anxiety or depression reported between December 2022 and December 2023. For an Intention to Treat (ITT) analysis of a stratified randomized trial, adjustment is recommended for the stratification variable^23,24^ on the principle that the analysis should follow the design^23,25^.

To assess the effect of the intervention according to symptoms of anxiety or depression, we restricted them to subsets with or without symptoms. The stratified models included only randomization arms as the independent variable. Similarly, post-hoc stratification analyses for the primary and secondary outcomes were conducted separately among subsets of participants based on the presence or absence of the post-hoc stratification factors (susceptibility to severe COVID-19, worry about COVID-19 and pre-trial perceptions of vaccine efficacy). The model included randomization arms and accounted for the presence or absence of symptoms of anxiety or depression.

Lastly, the validity of an ITT effect estimate requires correct adjustment for selection bias due to differential loss-to-follow-up and missing outcome data^23,26,27^. We used multiple imputation for loss-to-follow-up and missing data, hence conducting stratified analyses to assess effect modification rather than relying on interaction terms, as standard interaction testing isn’t directly applicable to imputed datasets^28^. Details on imputation methods are included in Appendix C. Methods for sensitivity analyses can be found in Appendix B. A series of sensitivity analyses were conducted to address assumptions related to loss to follow-up and protocol adherence, including complete case analysis, inverse probability weighting, per-protocol analysis for participants fully adhering to the intervention, and multiple imputation for missing outcomes, with consistent results observed across primary and secondary outcomes.

## Results

### Study Sample

Overall, 2,411 CHASING COVID Cohort participants were invited, and 1,419 (59%) were eligible and randomized to the intervention. After completing the 4-week survey, 16 participants were removed from the sample based on data quality protocols, leaving an analytic sample of 1403 participants. Randomization resulted in 469 individuals in CBT-kernels arm, 466 in the Inoculation arm, and 468 in the Standard public health messaging arm (Figure 1). Briefly, a majority of participants were female (56%), White non-Hispanic (60%), had at least a college education (58%), were employed (72%), and had employer-based health insurance (56%). Most (66%) did not report having any comorbidities. The median age of participants was 41, with 17% aged 60 or older. As of December 2023, participants reported a median of three COVID-19 vaccine doses, 74% reported having a primary care provider, and 38% had moderate-to-severe symptoms of anxiety or depression. Participants were balanced between groups on nearly all measured characteristics (SMD <0.2) except for reported discrimination in a healthcare experience (SMD=0.235). Prior to the intervention, a majority (70%) of participants were willing or very willing to get another COVID-19 vaccine dose, with differences in vaccine willingness across arms. Loss to follow-up was minimal [N = 56 (4%)] and did not differ by arm (p>0.9).

### Post-Intervention Perceptions

Immediately following the intervention, participants were asked if the video held their attention, with no significant differences across arms (p=0.6). 10% of participants reported they were very likely, 19% were somewhat likely, and 58% were unlikely to make time to get vaccinated, with no significant differences across arms (p=0.86). Immediately post-intervention, 16% of participants indicated they were planning to make a vaccine appointment, with no observed difference between arms (p=0.85). At four-week follow up, the same patterns held for willingness to make time to get vaccinated and likelihood to make a vaccine appointment. Additionally, the majority of participants (87%) reported no barriers to making a vaccine appointment, with no distinct differences between arms (p=0.4). Details are reflected in Appendix E.

### Primary Outcome: Vaccination at 4- weeks

The total number of vaccinations by 4-week follow up was low (N:17/1403, 1.2%, 95%CI: 0.6%-1.8%) and did not significantly differ by arm: 1.6% [N:7/469, 95%CI: 0.4%-2.8%] in the CBT-kernels arm, 0.9% [N:4/466, 95%CI: 0.0%-1.8] in the Inoculation arm, and 1.3% [N:6/468, 95%CI: 0.3%-2.4%] in the Standard arm (Table 1). There were no differences in vaccination among those with and without anxiety or depression symptoms. Reported COVID-19 infections were balanced across arms.

### Secondary Outcome: Vaccine Willingness at 4- weeks

For the secondary outcome, approximately one-third of respondents in each arm reported being very willing to get another dose of the COVID-19 vaccine at baseline: 30.7% in the CBT-kernels arm, 38.0% in the Inoculation arm, and 31.0% in the Standard arm (Table 2). Compared with the Standard arm, there were no significant differences in willingness across arms at 4-week follow-up: participants in the CBT-kernels arm were 0.2% less willing to vaccinate than those in the Standard arm (Risk Difference (RD): −0.2%, CI:−6.2%-5.9%; Risk Ratio (RR): 99.6%, CI:78.7%-126.0%), and participants in the Inoculation arm were 6.9% more willing to vaccinate than those in the Standard arm (RD: 6.9%, CI:0.7%-13.1%; RR: 122.4%, CI:97.8%-153.2%). There were no differences in vaccine willingness at 4-week follow-up when comparing those with and without anxiety or depression symptoms.

### Post-hoc Stratification Analyses of Primary and Secondary Outcomes.

There were no significant differences by age, worry about COVID-19, or pre-trial perceptions of vaccine efficacy for the primary outcome. However, individuals who were worried about COVID-19 and were in the Inoculation arm had higher vaccine willingness compared to controls (RR: 1.36, CI:1.04–1.79). There were no significant differences by age or pre-trial perceptions of vaccine efficacy for the secondary outcome.

### Sensitivity Analysis

The sensitivity analysis results (Supplemental Table 1, 2, 3) were largely consistent with the primary analysis (Tables 1 and 2), demonstrating similar vaccine uptake and willingness across intervention arms. The 4-week vaccine uptake rates remained stable, with minimal differences in risk ratios and risk differences between the two analyses. For instance, the risk ratio for vaccine uptake in CBT vs. Standard arm and Inoculation vs. Standard arm was 1.17 (95%CI: 0.39–3.49) and 0.68 (95%CI: 0.19–2.40), respectively in the sensitivity analysis (Supplemental Table 1), closely aligning with 1.21 (95%CI: 0.41–3.59) and 0.69 (95%CI: 0.19 – 2.44) in the primary analysis. Similarly, vaccine willingness in CBT vs. Standard arm and Inoculation vs. Standard arm showed a risk ratio of 1.00 (95%CI: 0.79–1.26) and 1.22 (95%CI: 0.98–1.53), respectively in the sensitivity analysis compared to 0.99 (95%CI: 0.785 – 1.25) and 1.23 (95%CI: 0.98–1.53) in the primary analysis. Minor differences in confidence intervals and complete case counts were observed, likely due to variations in handling missing data, but they did not materially affect the conclusions. These findings supported the robustness of the primary analysis and confirmed that the intervention effects remained stable across different analytical approaches.

## Discussion

This randomized controlled trial tested two novel COVID-19 vaccine messaging strategies—CBT-kernels and inoculation theory—against standard public health messaging among individuals who had received at least one prior dose and were eligible for another. We found no significant differences in vaccine uptake or willingness across arms, including among participants with or without mental health symptoms. These findings underscore the difficulty of influencing vaccine uptake, even among previously vaccinated individuals and those at higher risk for severe outcomes.

Inoculation theory-based messaging has rarely been evaluated in RCTs for vaccine promotion^4,5^, though observational studies suggest it may reduce receptivity to misinformation, a possible pathway to greater vaccine willingness^14^. Only one similar trial has tested this approach for vaccine uptake, with inconclusive findings, underscoring the need for further research^4,29^. Given our study took place years into the pandemic, the utility of inoculation messaging may be limited. The approach is designed to preempt misinformation before it takes hold—but by this stage, most individuals had already formed opinions about the vaccine. While our team identified a prevailing misinformation narrative related to vaccine effectiveness and used it to guide messaging^17^, entrenched beliefs may require more tailored strategies. Still, post-hoc stratification analyses among those worried about COVID-19 showed that inoculation messaging may hold promise to increase vaccine willingness. Follow-up at six months will further explore its impact on vaccine uptake.

To our knowledge, this is the first study to develop and test vaccine uptake using CBT-kernels. Several studies assessed the efficacy of CBT interventions on common mental health symptomatology during the pandemic^30,31^ and advocated for simultaneous evidence-based mental health treatment with COVID-19 public health messaging^16^; however, to our knowledge, the use of CBT-kernels to encourage adherence to COVID-19 prevention measures among individuals with common mental health symptoms has not been explored. Though we did not identify differences in vaccine uptake according to anxiety or depression symptomatology, even among those who received the CBT arm, this approach to developing non-symptom-related public health messaging for individuals with common mental health symptoms is novel and should be explored further. Future research should refine this approach to public health messaging by employing motivational interviewing strategies^32,33^ during the pilot testing phase to improve the messaging and effectiveness of such strategies.

Three large (N>100,000) RCTs grounded in ‘nudge theory’ examined the effects of SMS text reminders on vaccine uptake. Two found small but statistically significant increases in SARS-CoV-2 vaccines^4,34^ while another large trial did not^35^. Those trials that did find an effect observed 1–2% absolute increases in SARS-CoV-2 vaccine uptake from low levels at baseline. The framing of messaging was examined in one of these trials, showing that messages framed around ownership (e.g., *your* vaccine is ready and waiting for you), providing a specific date, time and location are most effective. While encouraging, these trials did not identify strategies capable of large absolute increases in COVID-19 vaccination among U.S. adults.

### Study Limitations

Our study was conducted outside the traditional respiratory virus season in the United States, well after the latest vaccines became available and prior to a significant summer COVID-19 wave. This was a period of relatively low COVID-19 risk: during the fielding period, every jurisdiction in the country reported low levels of respiratory illness activity, and most key indicators of COVID-19 activity nationally remained minimal^36,37^. This may have influenced the limited vaccine uptake among participants. Future follow-up is planned for approximately November 2024, coinciding with the release of the 2024 vaccine formulations. This will provide an opportunity to evaluate any long-term effects of intervention exposure. Additionally, given the study was conducted several years into the pandemic, the interaction of anxiety and depression and COVID-19 vaccination may differ from that observed at earlier stages of the pandemic.

Further, there are limitations to the interventions themselves. While video length was kept consistent across arms to maintain attention and align with typical media formats, this brevity may have constrained the ability to fully distinguish between communication approaches. Longer content might have enhanced the impact of each strategy. Participants may also have responded differently to the information presented had the actors in the video been different. Finally, although participants were required to remain on the video page its duration before proceeding, the study team could not confirm whether they actually watched the videos.

## Conclusion

This randomized controlled trial tested two novel COVID-19 vaccine messaging strategies—CBT-kernels and Inoculation theory-based messaging—against standard public health messaging among individuals who had already received at least one COVID-19 vaccination. This study contributes to the literature by operationalizing and testing two theoretical messaging strategies, yet the results highlight the complexities of addressing vaccine hesitancy in a population with entrenched beliefs. Although Inoculation theory-based messaging showed some promise in subgroup analysis, particularly among individuals still concerned about COVID-19, further investigation is needed to tailor these messages more effectively. Similarly, while the CBT-kernels approach aimed to address mental health-related barriers to vaccine uptake, no significant impact was observed, suggesting that additional refinements— such as incorporating motivational interviewing techniques—may enhance the efficacy of this approach in future interventions. Ultimately, the findings suggest that existing messaging strategies may be insufficient to drive substantial changes in vaccine uptake, and emphasize the need for continued exploration of more targeted and personalized communication interventions. Future research should refine these approaches and assess their long-term effects on vaccine behavior.

Due to a shift in U.S. federal research priorities, the NIH grant that supported this work was terminated before the project could be completed. While we were able to complete the trial and collect 4-week outcome data, the termination has greatly constrained our ability to conduct additional planned analyses and follow-up work. Nonetheless, our findings underscore the urgent need for continued research on strategies to promote vaccine uptake in ways that can reduce severe disease and death, including reducing the impact of mis/disinformation with attention to those with mental health symptoms. Although our trial did not find meaningful differences in vaccine uptake between study arms at four weeks, testing these theory-informed approaches adds to the growing body of evidence about what may —and may not—be effective. Future research should continue to explore, refine, and rigorously evaluate communication strategies that address informational and behavioral barriers to vaccination, including long-term effects on vaccine behavior, especially in the face of persistent disinformation and declining trust in public health.

## Data Availability

The data and code that support the findings of this study are available from the corresponding author upon reasonable request.

## Highlights

- Conducted a randomized control trial comparing attitudinal inoculation messaging, CBT-kernels messaging, and standard public health messaging to promote vaccine uptake in a cohort of eligible adults who were partially vaccinated against COVID-19.
- No significant differences in vaccine uptake or willingness to vaccinate were observed across the intervention groups at four-week follow up, highlighting the challenge of using theory-based messaging to increase vaccine uptake.
